# Auditing the Management of Vaccine-Preventable Disease Outbreaks: The Need for a Tool

**DOI:** 10.1371/journal.pone.0015699

**Published:** 2011-01-13

**Authors:** Nuria Torner, Dolors Carnicer-Pont, Jesus Castilla, Joan Cayla, Pere Godoy, Angela Dominguez, the Epidemiological Surveillance Working Group of Catalonia and Navarre, Spain

**Affiliations:** 1 Department of Health, Generalitat of Catalonia, Barcelona, Spain; 2 CIBER Epidemiologia y Salud Pública (CIBERESP), Barcelona, Spain; 3 Public Health Agency of Barcelona, Barcelona, Spain; 4 Public Health Institute of Navarre, Pamplona, Spain; 5 Department of Public Health, University of Barcelona, Barcelona, Spain; University of Liverpool, United Kingdom

## Abstract

Public health activities, especially infectious disease control, depend on effective teamwork. We present the results of a pilot audit questionnaire aimed at assessing the quality of public health services in the management of VPD outbreaks. Audit questionnaire with three main areas indicators (structure, process and results) was developed. Guidelines were set and each indicator was assessed by three auditors. Differences in indicator scores according to median size of outbreaks were determined by ANOVA (significance at p≤0.05). Of 154 outbreaks; eighteen indicators had a satisfactory mean score, indicator “updated guidelines” and “timely reporting” had a poor mean score (2.84±106 and 2.44±1.67, respectively). Statistically significant differences were found according to outbreak size, in the indicators “availability of guidelines/protocol updated less than 3 years ago” (p = 0.03) and **“**days needed for outbreak control” (p = 0.04). Improving availability of updated guidelines, enhancing timely reporting and adequate recording of control procedures taken is needed to allow for management assessment and improvement.

## Introduction

The mission of public health systems is to promote and protect the health of the population. One of the main facets of public health is to evaluate the effectiveness, accessibility and quality assessment of health services in the promotion and deliverance of good public health [1]. Most public health activities, and especially infectious disease control, depend on effective teamwork. Although assessment of quality of care by a public health audit is often perceived as difficult to accomplish, methods for quantitative quality assessment can aid this task [2]. An audit is concerned with ensuring that what is done is done right [3]. The terms used in the literature to define quality assessment and quality performance are often not consistently applied. We understand an audit as a quality assessment tool to measure the achievement of public health objectives and practices whereas quality performance would not only include quality but also efficiency. This study deals with quality assessment and does not measure costs. Public health quality indicators are statements on the capacity (structure), actions (processes) and results (outcomes) of public health practices [2]; [4].

Incorporating quality measurements into public health practice can be challenging due to the scarcity of background theory, research, evidence-based standards and practical experience from which to draw and develop useful indicators [2]. Indicators addressing the delivery of public health services can be developed to fill gaps in quality improvement efforts. However implementation of quality assessment is difficult due to limited detailed description of practices and sparse data resources [5]. The ultimate goal of quality measurement is to improve health outcomes by stimulating improvements in health care.Increasing recognition of deficiencies in quality is a spur to actions to improve health outcomes [6]. Therefore, a valid and reliable instrument for measuring public health quality assessment is an essential element of any attempt to examine public health practice [7].

Vaccine preventable disease (VPD) outbreaks are a major public health issue that requires immediate effective response including coordination of the different disciplines involved in their management.**** In the context of outbreaks, an audit would need to be an integral part of the process in order to ascertain satisfactory conclusions by means of evaluating explicit criteria based on relevant aspects of structure, processes and outcomes of outbreak management (investigation and control) [8].

Like other authors [9], we found few studies regarding this issue, with no comprehensive audit standards for outbreaks of VPD or communicable diseases being available. The investigation and control of VPD outbreaks should be evidence based. Guidelines and specific protocols have two main aims: a) to permit the application of experience in the setting of the outbreak or other settings and b) to make interventions applicable across settings in the entire territory or country.

An audit should be seen as an improvement tool for reviewing services delivered against explicit guidelines, identifying and implementing the necessary changes [10]. In this article, practical issues regarding the development of a set of public health quality indicators are described and discussed. The objective of this study is to present the results of a pilot questionnaire aimed at assessing the quality of public health services in the management of VPD outbreaks.

## Methods

Reports from the investigation of VPD outbreaks in the Catalonia and Navarre regions of Spain between 2003 and 2006 were studied. Data were collected and analyzed in 2008. An audit questionnaire with 21 key indicators for three main areas was developed by a group of epidemiologists who reached a consensus as to the structure and variables to assess. The criteria used were in accordance with Donabedian's framework, which divides quality into three dimensions: 1) structural quality assessing organizational features and resources available to manage the outbreak; 2) process quality assessing technical excellence and interaction with other disciplines and 3) outcome quality which assesses the influence of public health actions on outbreak control [4]. Indicators for each group are listed in Table S1. Scoring guidelines were agreed upon by consensus of all authors and each indicator was assessed by three auditors. In order to minimize inter-observational variation, the three researchers acting as auditors agreed upon individual indicator scores given. Quality and quantity values were scored on a Likert scale from 5 to 1 (5 =  Fully satisfactory; 4 = Satisfactory; 3 =  Acceptable; 2 = Poor; 1 = Unsatisfactory). Outbreaks were divided into two groups according to median value for outbreak size, considering outbreaks with less than four cases and those with four or more cases. Differences between medians were determined by the non-parametric Kruskal-Wallis test and differences between indicators according to size of outbreaks (<4 and ≥4 cases) were determined by ANOVA. The level of statistical significance was established as p≤0.05.

Information contained in final outbreak reports was studied to determine which variables could be included in a data base to carry out an explorative assessment of outbreak management performance. The variables included were size of the outbreak, attack rate, date of onset of symptoms of the first case, date of reporting to the surveillance unit in charge of the outbreak management. The existence of multidisciplinary teams or activity to control the outbreak, diffusion of information to all levels involved (citizens, health professionals, health authorities), median days required for outbreak control, the number of vaccines and immunoglobulins administered and the effectiveness of preventive measures applied were investigated. When required structural information was not available (indicators S1-S6) in the final reports, data were sought by contacting outbreak control teams directly.

## Results

After a consensus process for the development of a questionnaire with 21 indicators to audit the management of VPD outbreaks among the authors this has been tested over 154 VPD outbreaks. During the study period, 251 VPD outbreaks were recorded in the Catalonia and Navarre regions. Of these, 154 (61.3%) had reports containing sufficient information to be included in the study. The median outbreak size of was 3 cases (range 2-3056; SD±247.6). There were two outbreaks with more than 300 cases, 71% (109) had <4 cases and 10% (16) had >10 cases, with the median size value for large outbreaks being 19 cases (range 11-3056; SD±758.1). The greatest number of outbreaks were due to the hepatitis A virus and *Bordetella pertussis* with 62 outbreaks (43%) each, while measles, rubella and meningococcal disease accounted for <2% each. Table 1 shows that there was a statistically significant difference in the median size of outbreaks according to the etiology (p = 0.012).

**Table 1:** Median size of vaccine preventable disease outbreaks according to etiology.

Disease	Number of outbreaks (%)	Median size (range; SD) a
**Meningococcal Disease B**	2 (1.3)	2 (2-2;±0)
**Hepatitis A**	62 (40.3)	2 (2-49;±6.8)
**Hepatitis B**	5 (3.2)	2(2-11;±4.0)
**Mumps**	11 (7.1)	3 (2-3056; ±919.2)
**Rubella**	3 (1.9)	4(2-8; ±3.1)
**Measles**	3 (1.9)	7(3-381; ±217.1)
**Whooping cough**	62 (40.3)	3(2-11; ±1.9)
**Varicella**	6 (3.8)	14(3-49; ±17.8)
**Total**	154 (100)	3 (2-3056; ±247,6)

a Kruskal-Wallis p = 0.012.

Assessment of the audit questionnaire showed 16 indicators had a completely satisfactory or satisfactory mean score (4.27±1.42 to 5±0.0). One structure indicator (S1 “updated guidelines”), and one procedure indicator (P7 “timely reporting”) had a poor mean score (2.84±1.06 and 2.44±1.67, respectively). Process indicator P9, “daily recording of procedures”, had an acceptable score (3.09±0.75). According to outbreak size (<4 and ≥4 cases), there were differences in structure indicator S1, “availability of guidelines/protocol updated less than 3 years ago” (p = 0.03) and result indicator R17, **“**days needed for outbreak control” (p = 0.04) (Table S2).

## Discussion

A formal audit requires criteria upon which to base standards for good practice which should be in agreement with all concerned. The imposition of unacceptable external criteria for professionals involved will negate the purpose of the audit. In this study, consensus was attained among the members of the Epidemiologic Surveillance Working Group of Catalonia and Navarre in order to assess the quality of available retrospective information and to feedback results on the scope and content of future audit tools to be agreed upon by consensus among public health professionals involved and put into practice [11]. This study was a first step in the development of an audit questionnaire to improve the management of VPD outbreaks. There is evidence that auditing of patient records, for example, combined with discussion about improvements is one way to improve the quality of records and to change certain behaviors of healthcare professionals and it makes comparisons possible over time provided that a reliable audit instrument is used to put a numerical value on a written content [12].

According to Scutchfield et al, there are major areas of concern that must be addressed, such as data collection on structure, process and results, and standardization of data regardless of where or by whom it is collected so that it is well understood by all public health staff involved [7]. Likewise, we highlight the need to collect accurate VPD outbreak data, procedures carried out for outbreak control and outcomes in an understandable manner, according to timely updated guidelines or protocols for each VPD and using consensus variables for the auditing of VPD outbreak management.

Updated guidelines and protocols giving written instructions on how to manage a specific outbreak are crucial because staff with the knowledge may be unavailable at time of the outbreak onset [13].

In our study, structure indicator S1, “availability of guidelines/protocol updated less than 3 years ago”, scored poorly (2.84±1.06). In addition there were differences in the score for this structure indicator according to wether outbreaks had <4 cases or ≥4 cases. This may be because the greatest proportion of outbreaks were due to the hepatitis A virus and *Bordetella pertussis*, with a median outbreak size below 4 cases and with a lack of updated guidelines during the study period. The fact that the result indicators R17 “days needed for outbreak control” scored significantly higher for outbreaks with <4 cases (p = 0.04), meaning the more rapid outbreak control may be explained by the highly transmissible nature of the larger outbreaks (measles and varicella) (Tables 1 and S2).

Procedure indicator P7, “timely reporting”, had a poor mean score (2.44±1.67) suggesting that quality reporting should be enhanced. Trepka et al. found that increasing relationships between clinicians and public health staff results in an increased percentage of reported cases and improved reporting timelines. Other measures, such as shortening the list of reportable diseases for clinicians to those requiring contact investigations or immediate control efforts, such as VPDs, might increase compliance [14].

Process indicator P9 “daily recording of procedures” had an acceptable (3.09±0.75) because overall outbreak control activities were described in the final outbreak report. However, there was no outbreak with a dailyrecord management procedures was maintained. Standards of record-keeping may be more or less easy to set, but staff should be aware of the importance of good documentation in permitting correct auditing of processes at any time. In fact, only 61% of the VPD outbreaks occurring during the study period could be included because, in the remaining 39%, no detailed information was available to answer requested audit items correctly.

One limitation of the study was the difficulty in obtaining data from reports that are unevenly drawn up by surveillance units. These differences may partially be explained by a lack of computerized records of outbreak control procedures. [15]. In addition information was extracted by only three researchers who were in agreement to complete the questionnaires, raising doubts about the consistency of the study and whether the results can be extrapolated to other external auditors. However, as this was a pilot audit, we believe this does not invalidate the conclusions.Effective audit requires agreed criteria and standards considered suitable focusing upon the objective of enhancing quality service [11]. Johnston et al. found that it is possible to develop public health quality indicators and derive a quality ranking index for practice providing a comprehensive framework to encourage appraisal of current practice, identifying areas where change can be implemented [16].

Our study investigated the relevant issues regarding VPD outbreak data collection and studied the inferences that can be made from them in order to implement this tool for everyday practice. Subsequent auditing can also provide a means of measuring the effects of changes on quality of practice improvement and to recognize the need to make effective use of audit resources [16]; [17].

Some research groups and national outbreak managers [18]; [19], are leading initiatives to priorize of pathogens for surveillance and assess clinical governance in public health [20]. This would help to allocate resources for research and surveillance in public health at all levels (local, regional and national). The outcomes of these studies may provide relevant additional information and should be followed up considering their applicability to our experience.

Several studies have demonstrated that the adoption of electronic health records (EHR) can promote the quality of health care by reducing adverse events and improving management [21]; [22] in contrast with Keyhani et al. [23] who conclude that further research on how EHR are implemented and how they will improve the understanding of theirimpact on the quality of care are needed. Other studies highlight the importance of electronic reporting systems in improving the timeliness and completeness of reporting notifiable diseases [24]. We believe that the implementation of electronic systems for recording outbreaks would improve both reporting and evaluation of outbreak management. Early reporting of outbreaks of lower respiratory tract infections to local public health authorities was set up in France in 2006 to reduce associated morbidity and mortality. Reporting creates a dialog between nursing homes and public health professionals which facilitates outbreak management [25]. A link between surveillance units and laboratories has been shown to be positive in foodborne disease outbreaks. These include the Foodborne Diseases Active Surveillance Network (FoodNet), a collaborative project of the Centers for Disease Control and Prevention and the International Surveillance Network for the Enteric Infections Enter-net. This system captures microbiological confirmation of salmonella and verotoxinogenic *Escherichia coli* infections and has identified many international outbreaks allowing the implementation of public health interventions to prevent further cases. This system demonstrates that the dissemination of information on unusual events can lead to timely interventions. Similar actions could be also considered for VPD outbreaks.

Unconventional methods are currently being explored in order to attain early detection of contagious outbreaks. Relying on social network sensors allows for interventions such as vaccination of central individuals in networks that could enhance the population level efficacy [26].

In conclusion, we believe that the questionnaire used in this study containing structure, process and results indicators, although time consuming and not established as regular audit system, has proved its usefulness to audit outbreak management in our context. The purpose of this indicator system is to provide a tool that will enable quantitative quality assessment and feedback that will promote improvement in VPD outbreak management. A simple, automatic audit tool linked to an electronic reporting system, for regular and systematic use and assess its results should be considered [24]; [27]. Improving availability of updated guidelines, enhancing timely reporting and adequate recording of control procedures taken is needed to allow for management assessment and improvement.

## Supporting Information

Table S1 Indicator panels of the audit questionnaire for vaccine preventable disease outbreaks.
(DOC)

Table S2 Results of the three indicator panels' mean scores for vaccine preventable disease outbreaks.
(DOC)
