# Supplementary material for: Auditing the Management of Vaccine-Preventable Disease Outbreaks: The Need for a Tool
Source: PLoS One. 2011 Jan 13;6(1):e15699. doi: 10.1371/journal.pone.0015699 (PMC3020940; doi:10.1371/journal.pone.0015699)
Supplement: Table S1 — Indicator panels of the audit questionnaire for vaccine preventable disease outbreaks. (DOC) [file pone.0015699.s001.doc]

**Table S1. Indicator panels of the audit questionnaire for vaccine preventable disease outbreaks.**

| **Criteria** | | **Indicator and score level *** | **Data source** |
| --- | --- | --- | --- |
|  |  |  |  |
| **STRUCTURE INDICATORS** | | |  |
| **S1** |  | **Availability of guidelines/protocol updated less than 3 years ago** | **Guidelines** |
|  |  | 5= updated available; 3= available, but not updated; 1= Not available |  |
| **S2** |  | **Existence of multidisciplinary team for outbreak control** | **Report/OutbreakTeam** |
|  |  | A=Nursing staff, B= Epidemiologists; C= Clinician; D= Microbiologist; E = Media |  |
|  |  | In household outbreaks if A to D are involved score is considered 5 |  |
| **S3** |  | **Vaccine Availability** | **Report/OutbreakTeam** |
| **S4** |  | **Immunoglobulin Availability** | **Report/OutbreakTeam** |
| **S5** |  | **Nursing staff available for the administration of vaccines and immunoglobulins** | **Report/OutbreakTeam** |
| **S6** |  | **Communication procedure available** | **Report/OutbreakTeam** |
|  |  |  |  |
| **PROCESS INDICATORS** | | |  |
| **P7** |  | **Detection of the outbreak by correct reporting (health care professional)** | **Report/OutbreakTeam** |
|  |  | 5=Yes; 1 = Not available |  |
| **P8** |  | **Timely reporting** | **Report/OutbreakTeam** |
|  |  | On time: within 24 h of onset of first case symptoms |  |
|  |  | 5=≤24h ; 4=24-48h ; 3=48-72h ; 2= 4-15 days; 1=>15 days. |  |
|  |  | ***For HAV 1= >30days, 2=4-30days and for HBV 1= > 60 days, 2= 4-60 days |  |
| **P9** |  | **Daily recording of procedures “Outbreak log”** | **Report/OutbreakTeam** |
|  |  | 5= daily online registry ; 3=annotations in epidemiologic query form or in final report; 1=No | |
| **P10** |  | **Effective communication strategies implemented** | **Report/OutbreakTeam** |
|  |  | 5= Yes; 3= partially implemented; 1=No |  |
| **P11** |  | **Preventive outbreak control procedures set on time** | **Report/OutbreakTeam** |
|  |  | 5=<24h; 4=24-48h; 3= 48-72h; 2=3- 5 days; 1= > 15 days |  |
| **P12** |  | **Communication with partner agencies involved in the outbreak** | **Report/OutbreakTeam** |
| **P13** |  | **Communication procedures with those involved in the outbreak** | **Report/OutbreakTeam** |
|  |  | (informative meetings, letters, bulletins and briefs, media statements) |  |
| **P14** |  | **Setting a hypothesis on the nature and origin of the outbreak** | **Report/OutbreakTeam** |
| **P15** |  | **Case definition developed** |  |
| **P16** |  | **Implementation of explicit control measures adapted to the outbreak** | **Report/OutbreakTeam** |
|  |  |  |  |
| **RESULT INDICATORS** | | |  |
| **R17** |  | **Days needed for outbreak control** | **Guidelines** |
|  |  | Maximum incubation period *ratio to total duration of the outbreak |  |
| (number of incubation periods in the outbreak =generations) 5=1 generation; 4=2 - 4; 3=5 -7; 2=8-10; 1=>10 | | |  |
| *VHA=30 days; VHB = 60 days; MMR=21 days; Mumps = 25 days; Whooping cough= 20 days; Meningococcal disease= 10 days | | |  |
| **R18** |  | **Number of Vaccines administered** | **Report/OutbreakTeam** |
|  |  | Ratio needed/administered = 1 (100%) scores 5;75%=4;66%=3;25%=2;0%=1 |  |
| **R19** |  | **Number of Immunoglobulins administered** | **Report/OutbreakTeam** |
|  |  | Ratio needed/administered = 1 (100%) scores 5; 75%=4;66%=3;25%=2;0%=1 |  |
| **R20** |  | **Effectiveness of control measures** | **Report/OutbreakTeam** |
| **R21** |  | **Identification of the cause, source and mode of transmission** | **Report/OutbreakTeam** |
|  |  |  |  |
| *score level according to Likert scale (5= Fully satisfactory; 4=Satisfactory; 3= Acceptable; 2=Poor;1=Unsatisfactory | | |  |
