# Supplementary material for: Auditing the Management of Vaccine-Preventable Disease Outbreaks: The Need for a Tool
Source: PLoS One. 2011 Jan 13;6(1):e15699. doi: 10.1371/journal.pone.0015699 (PMC3020940; doi:10.1371/journal.pone.0015699)
Supplement: Table S2 — Results of the three indicator panels' mean scores for vaccine preventable disease outbreaks. (DOC) [file pone.0015699.s002.doc]

**Table S3**. Results of the three indicator panels’ mean scores for vaccine preventable disease outbreaks.

| **STRUCTURE INDICATORS** | | | **All outbreaks** | **Outbreaks with**  **<4 cases ≥4 cases** | | |  | |
| --- | --- | --- | --- | --- | --- | --- | --- | --- |
|  |  |  | **Mean Score (±SD)** | **Mean Score (±SD)** | **Mean Score (±SD) p** | | | |
| S1 | Availability of guidelines/protocol updated less than 3 years ago | | 2.84 (±1.06) | 2.96 (±0.94) | | 2.56 (±1.27) | | 0.030 |
| S2 | Existence of multidisciplinary team for outbreak control | | 4.97 (±0.32) | 5.00 (±0.00) | | 4.91 (±0.602) | | NS |
| S3 | Vaccine Availability | | 4.92 (±0.39) | 4.91 (±0.42) | | 4.96 (±0.30) | | NS |
| S4 | Immunoglobulin Availability | | 4.77 (±0.98) | 4.72 (±1.06) | | 4.87 (±0762) | | NS |
| S5 | Nursing staff available for the administration of vaccines and immunoglobulins | | 5.00 (±0.00) | 5.00 (±0.00) | | 5.00 (±0.00) | | NS |
| S6 | Communication procedure available | | 4.90 (±0.43) | 4.90 (±0.45) | | 4.89 (±0.38) | | NS |
| **PROCESS INDICATORS** | | |  |  | |  | |  |
| P7 | | Detection of the outbreak by correct reporting (health care professional) | 4.63 (±1.01) | 4.70 (±0.97) | | 4.47 (±1.33) | | NS |
| P8 | | Timely reporting | 2.44 (±1.67) | 2.60 (±1.69) | | 2.04 (±1.57) | | NS |
| P9 | | Daily recording of procedures “Outbreak log” | 3.09 (±0.75) | 3.09 (±0.77) | | 3.09 (±0.70) | | NS |
| P10 | | Effective communication strategies implemented | 4.76 (±0.93) | 4.72 (±1.02) | | 4.88 (±0.66) | | NS |
| P11 | | Preventive outbreak control procedures set on time | 4.27 (±1.42) | 4.29 (±1.43) | | 4.22 (±1.40) | | NS |
| P12 | | Communication with other partner agencies involved in the outbreak | 4.82 (±0.79) | 4.83 (±0.78) | | 4.82 (±0.83) | | NS |
| P13 | | Communication procedures with those involved in the outbreak | 4.73 (±1.02) | 4.72 (±1.03) | | 4.73 (±1.01) | | NS |
| P14 | | Setting a hypothesis on the nature and origin of the outbreak | 4.86 (±0.72) | 4.85 (±0.76) | | 4.98 (±0.61) | | NS |
| P15 | | Case definition developed | 4.86 (±0.72) | 4.89 (±0.65) | | 4.80 (±0.84) | | NS |
| P16 | | Implementation of explicit control measures adapted to the outbreak | 4.65 (±1.08) | 4.65 (±1.06) | | 4.64 (±1.15) | | NS |
| **RESULT INDICATORS** | | |  |  | |  | |  |
| R17 | | Days needed for outbreak control | 4.36 (±1.21) | 4.49 (±1.10) | | 4.05 (±1.40) | | 0.04 |
| R18 | | Number of vaccines administered | 4.67 (±1.14) | 4.65 (±1.16) | | 4.71 (±1.10) | | NS |
| R19 | | Number of immunoglobulins administered | 4.51 (±1.41) | 4.50 (±1.41) | | 4.51 (±1.40) | | NS |
| R20 | | Effectiveness of control measures | 4.60 (±1.10) | 4.63 (±1.06) | | 4.52 (±1.19) | | NS |
| R21 | | Identification of the cause, source and mode of transmission | 4.64 (±1.11) | 4.63 (±1.13) | | 4.64 (±1.06) | | NS |
